# Supplementary material for: Neuroinflammation Is Associated with GFAP and sTREM2 Levels in Multiple Sclerosis
Source: Biomolecules. 2022 Jan 27;12(2):222. doi: 10.3390/biom12020222 (PMC8961656; doi:10.3390/biom12020222)
Supplement: Supplementary file 1 [file biomolecules-12-00222-s001.zip › biomolecules-1509343-supplementary.pdf]

Supplementary Materials

Table S1: Correlations between GFAP/TREM2 and CSF inflammatory molecules

GFAP	R	*p*	B-H adjusted *p*	sTREM2	R	*p*	B-H adjusted *p*
**sTREM2**	**0.648**	**<0.001**	**<0.001**	GFAP	-	-	-
IL-1	0.921	0.519	0.628	IL-1	0.143	0.485	0.587
IL-2	−0.129	0.367	0.543	IL-2	−0.172	0.233	0.412
IL-4	0.111	0.439	0.578	IL-4	0.247	0.081	0.155
**IL-5**	**0.432**	**0.002**	**0.009**	**IL-5**	**0.397**	**0.004**	**0.018**
IL-6	−0.126	0.378	0.543	IL-6	−0.051	0.722	0.788
IL-7	0.243	0.086	0.188	IL-7	0.251	0.076	0.155
**IL-8**	**0.495**	**<0.001**	**0.002**	**IL-8**	**0.431**	**0.002**	**0.011**
IL-9	0.312	0.026	0.099	**IL-9**	**0.428**	**0.002**	**0.011**
IL-10	0.057	0.693	0.755	IL-10	0.113	0.443	0.566
IL-12	0.108	0.452	0.578	IL-12	0.123	0.389	0.526
IL-13	0.267	0.058	0.167	**IL-13**	**0.368**	**0.008**	**0.031**
IL-15	0.011	0.937	0.937	IL-15	0.014	0.921	0.921
IL-17	0.182	0.206	0.364	IL-17	0.138	0.333	0.495
**G-CSF**	**0.576**	**<0.001**	**0.001**	**G-CSF**	**0.576**	**<0.001**	**0.001**
GM-CSF	−0.169	0.236	0.388	GM-CSF	−0.069	0.632	0.726
MCP1	0.072	0.617	0.709	MCP1	−0.029	0.839	0.877
RANTES	0.275	0.047	0.164	RANTES	0.257	0.069	0.155
MIP1A	0.138	0.845	0.883	MIP1A	0.294	0.036	0.118
TNF	0.244	0.084	0.188	TNF	0.135	0.345	0.496
**IFNγ**	**0.432**	**0.002**	**0.009**	IFNγ	0.261	0.065	0.155
IL-1ra	0.185	0.194	0.364	IL-1ra	0.247	0.082	0.155
IP10	0.243	0.087	0.188	IP10	0.162	0.256	0.421

Abbreviations. R: Spearman's rho; *p*: *p*-value, B-H adjusted *p*: Benjamini- Hochberg adjusted *p*-value; GFAP: glial fibrillary acidic protein, sTREM2: soluble triggering receptor expressed on myeloid cells-2, IL: interleukin; G-CSF: granulocyte colony stimulating factor, GM-CSF: granulocyte macrophage colony stimulating factor; MCP1: Monocyte chemoattractant protein-1; RANTES: Regulated upon Activation, Normal T Cell Expressed and Presumably Secreted; MIP1A: Macrophage Inflammatory Proteins 1A; TNF: tumor necrosis factor; INF: interferon, ra: receptor antagonist; IP10: Interferon gamma-induced protein 10
